# Supplementary material for: Assessments of and Attitudes towards Specialist Teleconsultations among Nephrology and Posttransplant Outpatients during the COVID-19 Pandemic
Source: Healthcare (Basel). 2023 Oct 14;11(20):2737. doi: 10.3390/healthcare11202737 (PMC10606026; doi:10.3390/healthcare11202737)
Supplement: Supplementary file 1 [file healthcare-11-02737-s001.zip › healthcare-2607868-supplementary.pdf]

The assessment of and attitude towards specialist teleconsultations among patients of Nephrology and Posttransplant Outpatient Clinics

The survey is anonymous and should not be signed.

The survey consists of 32 closed questions and 1 open question.

The survey takes about 5 minutes to complete.

In each closed-ended question, please mark 1 answer, unless the instructions say otherwise.

0. Introduction information

0.1. Age ________ years

0.2. Gender ☐ F / ☐ M

0.3. Education

☐ Elementary/high school
☐ Elementary vocational
☐ Secondary (high school, technical school)
☐ Higher

0.4. Status

☐ Person studying
☐ Person working
☐ Person not working
☐ Pensioner
☐ Person receiving benefits

0.5. Place of residence

☐ Countryside
☐ City up to 10 thousand residents
☐ City of 10 – 50 thousand residents
☐ City of 50 – 100 thousand residents
☐ City of 100 – 500 thousand residents
☐ City of more than 500 thousand residents

0.6. Do you use remote services e.g.: virtual access to the office/mobile banking/online shopping?

☐ YES ☐ NO

0.7. Do you use an Internet Patient Account?

☐ YES ☐ NO

1. Using of teleconsultations

1.1. Have you ever used teleconsultation in a nephrology clinic or after a kidney transplant?

☐ YES ☐ NO

YES then please complete the following questions **NO** then go to question1.2:

1.1.1. How many times have you used teleconsultation at a nephrology outpatient clinic or after kidney transplantation in the past 2 years?

☐ 1 ☐ 2-4 ☐ 5 or more

1.1.2. Did you have a choice between a teleconsultation or an face to face visit?

☐ YES ☐ NO

1. 1. 3. How long had you been a member of a nephrology or renal transplant clinic when you used teleconsultation for the first time?

☐ < 6 months ☐ 6 months – 2 years ☐ 2-5 years ☐ > 5 years

1. 1. 4. What is the distance of your residence from a nephrology or renal transplant clinic?

☐ Same city ☐ < 10km ☐ 10-50 km ☐ > 50km

1.2. How many times have you used an inpatient visit to a nephrology clinic or after a kidney transplant in the past 2 years?

☐ 0 ☐ 1 ☐ 2-4 ☐ 5 or more

1.3. How many other (besides nephrology or kidney transplant clinics) specialty clinics have you used in the past 2 years?

☐ None ☐ 1 ☐ 2 or more

1.4. Have you used teleconsultation at a family doctor?

☐ YES ☐ NO

YES then please complete the following questions **NO** then go to question 1.5:

1.4.1. How many times have you used teleconsultation in the family doctor in the last 2 years?

☐ 1 ☐ 2-4 ☐ 5 or more

1.4.2. Please rate your level of satisfaction with the health care services provided during teleconsultation at the family doctor

*Mark an **x** in the appropriate cell*

1<br>Negative assessment	1	2	3	4	5	5<br>Positive assessment

1.5. How many times have you used an inpatient visit to a family doctor in the past 2 years?

☐ 0 ☐ 1 ☐ 2-4 ☐ 5 or more

2. The course of teleconsultation in a nephrology or transplant clinic

2.1. In what form was the teleconsultation conducted?

☐ Telephone consultation ☐ Email ☐ Videoconversation

2.2. What was the purpose of the teleconsultation(s)?
*Please mark **ALL** the appropriate ones*

☐ Renewal of prescriptions ☐ Discussion of test results
☐ Follow-up visit/plan ☐ Consultation due to the new symptoms
☐ Other: ____

2.3. Was the information provided during the teleconsultation understood by you?

☐ Definitely yes ☐ Rather yes ☐ Hard to say
☐ Rather not ☐ Definitely not

2.4. Did you need help from loved ones during the teleconsultation?

☐ YES ☐ NO

2.5. Has the problem you addressed been resolved?

☐ YES ☐ NO

YES then go to question 3.1 **NO** then complete the following question

2.5.1 In a situation where the teleconsultation did not fully solve the problem, was there an opportunity to make an in-person appointment?

☐ YES ☐ NO

3. Attitudes toward teleconsultation

3.1. What do you think are the main advantages of telemedicine?
*Please mark **ALL** the appropriate ones*

☐ Ability to consult without leaving home
☐ Less risk of infection
☐ Shorter waiting time for advice
☐ I don't see any advantages
☐ Other: ____

3.2. What do you think are the biggest disadvantages of telemedicine?
*Please mark **ALL** the appropriate ones*

☐ Difficulty in understanding the recommendations given by the doctor
☐ Lack of opportunity for the doctor to conduct an examination
☐ Technical problems during the telemedicine (e.g., interruption, interference, crackling)
☐ Problems in describing symptoms-worse understanding by the doctor
☐ Greater risk of misdiagnosis
☐ I don't see any defects
☐ Other: ____

3.3. Would you be willing to use teleconsultation in the future:

☐ Definitely yes ☐ Rather yes ☐ Hard to say
☐ Rather not ☐ Definitely not

3.4. Please rate your level of satisfaction with the health services provided during the teleconsultation at the nephrology/kidney transplant clinic:

*Mark an **x** in the appropriate cell*

1<br>Negative rating	1	2	3	4	5	5<br>Positive rating

3.5 In the following situations, do you prefer a remote or in-patient visit to a specialist clinic::

*Mark an **x** in the appropriate cell*

3.5.1. Renewal of prescriptions

Strongly prefer teleconsultation	Rather prefer teleconsultation	I don't see the advantage of either form of visit	Rather prefer in-patient visit	Strongly prefer in-patient visit

3.5.2. Discussion of laboratory results

Strongly prefer teleconsultation	Rather prefer teleconsultation	I don't see the advantage of either form of visit	Rather prefer in-patient visit	Strongly prefer in-patient visit

3.5.3. Follow-up/scheduled visit

Strongly prefer teleconsultation	Rather prefer teleconsultation	I don't see the advantage of either form of visit	Rather prefer in-patient visit	Strongly prefer in-patient visit

3.5.4. Consultation due to new symptoms

Strongly prefer teleconsultation	Rather prefer teleconsultation	I don't see the advantage of either form of visit	Rather prefer in-patient visit	Strongly prefer in-patient visit

3.6 Would you recommend a teleconsultation at a specialty clinic to a loved one?

*Mark an **x** in the appropriate cell*

0<br>Definitely not	0	1	2	3	4	5	6	7	8	9	10	10<br>Definitely yes

Thank you for completing the survey!
